# Asymptotics of Subsampling for Generalized Linear Regression Models under Unbounded Design

**DOI:** 10.3390/e25010084

**Published:** 2022-12-31

**Authors:** Guangqiang Teng, Boping Tian, Yuanyuan Zhang, Sheng Fu

**Affiliations:** 1 School of Mathematics, Harbin Institute of Technology, Harbin 150001, China; 2 School of Mathematical Sciences, Soochow University, Suzhou 215006, China; 3 Department of Industrial Systems Engineering & Management, National University of Singapore, 21 Lowr Kent Ridge Road, Singapore 119077, Singapore

**Keywords:** generalized linear models, massive data, nonnatural links, unbounded covariates, unconditional subsampling estimator

## Abstract

The optimal subsampling is an statistical methodology for generalized linear models (GLMs) to make inference quickly about parameter estimation in massive data regression. Existing literature only considers bounded covariates. In this paper, the asymptotic normality of the subsampling M-estimator based on the Fisher information matrix is obtained. Then, we study the asymptotic properties of subsampling estimators of unbounded GLMs with nonnatural links, including conditional asymptotic properties and unconditional asymptotic properties.

## 1. Introduction

In recent years, the amount of information that people need to process is increasing dramatically. It is of great challenge to directly process massive data for statistical analysis. The divide-and-conquer strategy can mitigate the challenge of directly processing such big data [1], but it still consumes considerable computing resources. As a cheaper alternative in computing, subsampling gains its value in the case of limited computing resources.

To reduce the burden on the machine, the subsampling strategy based on big data has been given more attention in recent years. Ref. [2] proposes simple necessary and sufficient conditions for a convolved subsampling estimator to produce a normal limit that matches the target of bootstrap estimation; Ref. [3] provides an optimally distributed subsampling for maximum quasi-likelihood estimators with massive data; Ref. [4] studies some adaptive optimal subsampling algorithms; and Ref. [5] describes a subdata selection method based on leverage scores which conduct the linear model selection on a small subdata set.

GLM is a kind of statistical model with a wide range of applications such as [6,7,8]. Many subsampling studies are based on GLMs such as [3,9,10]. However, the covariates of the subsampled GLMs in the literature are bounded. When dealing with some big data problems, the size of covariate is not strictly bounded, such as the number of clicks on a web page, which can grow infinitely. This requires the extension of existing theories to the unbounded design. To fill this gap, this paper aims to study asymptotic properties of the subsampled GLMs with unbounded covariates based on empirical process and martingale technology.

Our three contributions are shown as follows: (1) we describe the asymptotic property of subsampled M-estimator using Fisher information matrix; (2) we give the conditional consistency and asymptotic normality of unbounded GLMs subsampling estimator; (3) we provide the unconditional consistency and asymptotic normality of unbounded GLMs subsampling estimator.

The rest of the paper is organized as follows. Section 2 introduces the basic concepts in GLMs and subsampling M-estimation problem. Section 3 presents the asymptotical properties for unbounded GLMs subsampling estimators. Section 4 gives the conclusion and discussion, as well as future research directions. All the technical proofs are collected in the Appendix A.

## 2. Preliminaries

This section introduces the subsampling M-estimation problem and GLMs.

### 2.1. Subsampling M-Estimation

Let {l(β;Z)∈R|Z∈Z} be a set of loss functions with a finite dimensional convex set $\beta \in \Theta \subset R^{p}$, and U={1,2,…,N} be the index of the full large dataset with σ-algebra $F_{N}=\sigma \left(Z_{1},\ldots ,Z_{N}\right)$, where for each i∈U, the random data point $Z_{i}\in Z$ (some probability space) is observed. The empirical risk $L_{N}:\Theta \to R$ is given by $L_{N}\left(\beta \right)=\frac{1}{N}\sum_{i\in U}l\left(\beta ;Z_{i}\right)$.

The goal is to get the solution ${\hat{\beta }}_{N}$ to minimize the risk, namely

(1) $${\hat{\beta }}_{N}=\arg\min_{\beta \in \Theta }L_{N}\left(\beta \right).$$

To solve Equation (1), we need ${\hat{\beta }}_{N}$ satisfy: $\nabla L_{N}\left(\beta \right)=\frac{1}{N}\sum_{i\in U}\nabla l\left(\beta ;Z_{i}\right)=0$, and let $\Sigma_{N}:=\nabla^{2}L_{N}\left({\hat{\beta }}_{N}\right)$. This is an M-estimation problem; see [11]. For fast solving large-scale estimation in Equation (1), we propose the subsampling M-estimation. Consider an index set $S=\{i_{1},i_{2},\ldots ,i_{n}\}$ with replacement from *U* according to the sampling probability ${\left\{\pi_{i}\right\}}_{i=1}^{N}$ such that $\sum_{i=1}^{N}\pi_{i}=1.$ The subsampling M-estimation problem is to obtain the solution ${\hat{\beta }}_{n}$ satisfying

$$\nabla L_{n}\left(\beta \right)=0\;\;\mathrm{with}\;\;L_{n}\left(\beta \right)=\frac{1}{Nn}\sum_{i\in S}\frac{1}{\pi_{i}^{*}}l\left(\beta ;Z_{i}^{*}\right),$$

where $Z_{i}^{*}$ is the *i*-th time subsample with replacement and $\pi_{i}^{*}$ is the subsampling probability of $Z_{i}^{*}$. For example, if $Z_{1}^{*}=Z_{1}$, then $\pi_{1}^{*}=\pi_{1}$; if $Z_{2}^{*}=Z_{1}$, then $\pi_{2}^{*}=\pi_{1}$. Denote $a_{i}$ as the number of *i*-th subsampled data such that $\sum_{i\in U}a_{i}=n$. And $L_{n}\left(\beta \right)$ is constructed by inverse probability weighting skill such that $E\left[L_{n}\left(\beta \right)\right|F_{N}]=L_{N}\left(\beta \right)$; see [12]. Details about properties of conditional expectation are shown in [13].

### 2.2. Generalized Linear Models

Let the random variable *Y* be the distribution of the natural exponential families $P_{\alpha }$ indexed by parameter α,

(2) $$P_{\alpha }\left(dy\right)=dF_{Y}\left(y\right)=c\left(y\right)\exp\left\{y\alpha -b\left(\alpha \right)\right\}\nu \left(dy\right),c\left(y\right)>0,$$

where α is often referred to as the canonical parameter belonging to its natural space

Λ={α:∫c(y)exp{yα}ν(dy)<∞}.

ν(·) is the Lebesgue measure for continuous distributions (Normal, Gamma) or counting measure for discrete distributions (binomial, Poisson, negative binomial). The c(y) is free of α.

Let ${\left\{\left(Y_{i},X_{i}\right)\right\}}_{i=1}^{N}$ be *N* independent sample data pairs. Here the $X_{i}\in R^{p}$ is covariates and we assume that the response $Y_{i}$ follows the distribution of the natural exponential families with the parameter $\alpha_{i}\in \Lambda$. The covariates $X_{i}:={\left(x_{i1},\ldots ,x_{ip}\right)}^{T},\left(i=1,2,\ldots ,N\right)$ are supposed to be deterministic.

The conditional expectation of $Y_{i}$ for a given $X_{i}$ is defined as a function of $\beta^{T}X_{i}$ after a transformation by a link function $\alpha_{i}=\psi \left(\beta^{T}X_{i}\right)$. The mean value denoted as $\mu_{i}:=E\left(Y_{i}\right)$ is mostly considered for regression.

If $\alpha_{i}=\beta^{T}X_{i}$ then we call that $\psi \left(\beta^{T}X_{i}\right)=\beta^{T}X_{i}$ is canonical (or natural) link function, and corresponding model is canonical (or natural) GLMs; see Page 32 in [14]. Sometimes the assumption $\alpha_{i}=\beta^{T}X_{i}$ is somewhat strong and not very suitable in practice, while nonnatural link GLMs allow more flexible choices for the link function. We can further assume that $\alpha_{i}$ and $\beta^{T}X_{i}$ can be related by a nonnatural link function $\alpha_{i}=\psi \left(\beta^{T}X_{i}\right)$.

Let $f_{\beta }\left(Y_{i}|X_{i}\right)$ be the joint density function of the i.i.d. data ${\left\{\left(Y_{i},X_{i}\right)\right\}}_{i=1}^{N}$ from the exponential family with a link function ψ(·). Then the nonnatural GLMs [15] is defined by

(3) $$Y_{i}|X_{i}∼f_{\beta }\left(Y_{i}|X_{i}\right)=c\left(Y_{i}\right)\exp\left\{Y_{i}\psi \left(\beta^{T}X_{i}\right)-b\left(\psi \left(\beta^{T}X_{i}\right)\right)\right\},\;i=1,2,\ldots ,N.$$

Here is a classic result for the exponential family (3),

(4) $$E\left(Y_{i}|X_{i}\right):=\mu_{i}=\dot{b}\left(\alpha_{i}\right)=\dot{b}\left(\psi \left(\beta^{T}X_{i}\right)\right)\;and\;\mathrm{Var}\left(Y_{i}|X_{i}\right):=\mathrm{Var}\left(Y_{i}\right)=\ddot{b}\left(\alpha_{i}\right),$$

where i=1,2,…,N; see P280 in [16].

## 3. Main Results

### 3.1. Subsampling M-Estimation Problem

In this part we first look at the term $\Sigma_{N}^{-1}\nabla L_{n}\left({\hat{\beta }}_{N}\right)$. Define an independent random vector sequence ${\left\{\zeta_{j}\right\}}_{j=1}^{N}$ and the subsampled ${\left\{\zeta_{j}^{*}\right\}}_{j=1}^{n}$, such that each vector ζ takes the value among ${\left\{\frac{1}{N\pi_{i}}\Sigma_{N}^{-1}\nabla l\left({\hat{\beta }}_{N};Z_{i}\right)\right\}}_{i=1}^{N},$ and let

$$V_{M}\left({\hat{\beta }}_{N};n\right)=\frac{1}{N^{2}n}\Sigma_{N}^{-1}\left[\sum_{i\in U}\frac{1}{\pi_{i}}\nabla l\left({\hat{\beta }}_{N};Z_{i}\right)\nabla^{T}l\left({\hat{\beta }}_{N};Z_{i}\right)\right]\Sigma_{N}^{-1}.$$

From the definition of $\nabla L_{N}\left(\beta \right)$, we have $E\left(\zeta |F_{N}\right)=\Sigma^{-1}\nabla L_{N}\left({\hat{\beta }}_{N}\right)=0$ and $\mathrm{Var}(\zeta |F_{N})$$=nV_{M}\left({\hat{\beta }}_{N};n\right).$ Then we have the asymptotic property of subsampled M-estimator.

**Theorem** **1.** *Suppose that the risk function $L_{N}\left(\beta \right)$ is twice differentiable and λ-strongly convex over *Θ*, that is, for β∈Θ, $\nabla^{2}L_{N}\left(\beta \right)\geq \lambda I$, where ≥ denotes the semidefinite positive ordering; and the sampling-based moment condition,*

$$\begin{array}{c} \frac{1}{N^{4}}\sum_{i=1}^{N}\frac{1}{\pi_{i}^{3}}{∥\nabla l({\hat{\beta }}_{N};Z_{i})∥}^{4}=O_{P}\left(1\right). \end{array}$$

*Then we can obtain: As n→∞, conditioning on $F_{N}$,*

(5) $$V_{M}{\left({\hat{\beta }}_{N};n\right)}^{-\frac{1}{2}}\left({\hat{\beta }}_{n}-{\hat{\beta }}_{N}\right)\overset{d}{\to }N\left(0,I_{p}\right),$$

*where $\overset{d}{\to }$ means convergence in distribution.*

Theorem 1 reveals that the subsampling M-estimation scheme is theoretically feasible under mild conditions. In addition, the existence of the estimator is given by the Fisher information matrix.

### 3.2. Conditional Asymptotic Properties of Subsampled GLMs with Unbounded Covariates

The exponential family is very versatile for containing many common light-tail distributions such as binomial, Poisson, negative binomial, normal and Gamma. Along with their attendant convexity properties which leads to finite variance property for log-density, they can serve for a large amount of popular and effective statistical models. It is precisely because of the commonality of these distributions so that we study the subsampling problem for GLMs.

From the loss function introduced in Section 2.1, we set $l\left(\beta ;Z_{i}\right):=-\log f_{\beta }\left(Y_{i}|X_{i}\right)$ where $f_{\beta }\left(Y_{i}|X_{i}\right)$ is defined by Equation (2), then the problem solving the minimum of the loss function is equivalent to solve the maximum of the likelihood function. For simplicity, we assume that c(y)=1, then

$$\nabla l\left(\beta ;Z_{i}\right):=-\frac{\partial \log f_{\beta }\left(Y_{i}|X_{i}\right)}{\partial \beta }=-\left[Y_{i}-\dot{b}\left(\psi \left(\beta^{T}X_{i}\right)\right)\right]\dot{\psi }\left(\beta^{T}X_{i}\right)X_{i}$$

with the nonnatural link function $\alpha_{i}=\psi \left(\beta^{T}X_{i}\right)$. We also use this idea in Section 3.3.

More generally, we consider a wider class saying quasi-GLMs, rather than GLMs, which assumes that Equation (4) holds for a certain function μ(·). Strong consistency and asymptotic normality of quasi maximum likelihood estimate in GLMs with bounded covariates are proved in [17]. For unbounded covariates, adopting the subsampled estimation of GLMs in [9], we calculate the inverse probability weighted estimator of β by solving the estimating equation based on the subsampled index set *S*,

$$-\frac{1}{Nn}\sum_{i\in S}\frac{1}{\pi_{i}^{*}}\left[Y_{i}^{*}-\mu \left(\psi \left(\beta^{T}X_{i}^{*}\right)\right)\right]\dot{\psi }\left(\beta^{T}X_{i}^{*}\right)X_{i}^{*}=0.$$

where ${\left\{\left(Y_{i}^{*},X_{i}^{*}\right)\right\}}_{i\in S}$ is subsampled data. Equivalently, we have

(6) $$s_{n}\left(\beta \right)=\sum_{i\in S}\frac{1}{\pi_{i}^{*}}\left[Y_{i}^{*}-\mu \left(\psi \left(\beta^{T}X_{i}^{*}\right)\right)\right]\dot{\psi }\left(\beta^{T}X_{i}^{*}\right)X_{i}^{*}=0.$$

Equation (6) is called quasi-GLMs since Equation (4) is given instead of the distribution function.

Let ${\hat{\beta }}_{n}$ be the estimator of the real parameter $\beta_{0}$ in subsampled quasi-GLMs and ${\hat{\beta }}_{N}$ be the estimator of $\beta_{0}$ in quasi-GLMs with full data. For the unbounded quasi-GLMs with full data, ${\hat{\beta }}_{N}$ is asymptotic unbiased with respect to $\beta_{0}$; see [18]. Next, we focus on the asymptotical properties of ${\hat{\beta }}_{n}$, as shown in the following theorems.

**Theorem** **2.** *Let ${\left\{\left(Y_{i}^{*},X_{i}^{*}\right)\right\}}_{i\in S}$ be subsampled from i.i.d. full data ${\left\{\left(Y_{i},X_{i}\right)\right\}}_{i\in U}$. Consider the Equation (4) and (6) where ψ(·) is three times continuously differentiable whose every derivative is bounded, and b(·) is twice continuously differentiable whose every derivative is also bounded. Assume that:*

*(A.1)*  *The range of the unknown parameter β is an open subset of $R^{p}$.*
*(A.2)*  *For any i∈S, $E\sup_{\beta \in \Theta }\left[\frac{1}{\pi_{i}^{*}}\left|Y_{i}^{*}-\mu \left(\psi \left(\beta^{T}X_{i}^{*}\right)\right)\right||F_{N}\right]=O\left(1\right)$.*
*(A.3)*  *For any β∈Θ and i∈S, $0<\inf_{i}\varphi \left(\beta^{T}X_{i}^{*}\right)\leq \sup_{i}\varphi \left(\beta^{T}X_{i}^{*}\right)<\infty$, where $\varphi \left(t\right)={\left[\dot{\psi }\left(t\right)\right]}^{2}\ddot{b}\left(\psi \left(t\right)\right)$.*
*(A.4)*  *For any $\beta_{1}\in \Theta$ and $\beta_{2}\in \Theta$, there exists a function $|m(X_{i}^{*})|<\infty$ such that*
$$|\varphi \left(\beta_{1}^{T}X_{i}^{*}\right)-\varphi \left(\beta_{2}^{T}X_{i}^{*}\right)|\leq |m\left(X_{i}^{*}\right)\left|\right|\beta_{1}^{T}X_{i}^{*}-\beta_{2}^{T}X_{i}^{*}|.$$
*(A.5)*  *When $n\to \infty ,\max_{i\in S}X_{i}^{*T}{\left(X^{*}X^{*T}\right)}^{-1}X_{i}^{*}=O\left(n^{-1}\right)$ and $\lambda_{\min}\left[X^{*}X^{*T}\right]\to \infty$, where $X^{*}=\left(X_{1}^{*},...,X_{n}^{*}\right)$ and $\lambda_{\min}\left[A\right]$ is the smallest eigenvalue of the matrix A.*
*(A.6)*  *$\min_{i=1,...,N}\left(N\pi_{i}\right)=O\left(1\right)$, $\max_{i=1,...,N}\left(N\pi_{i}\right)=O\left(1\right)$.*

*Then ${\hat{\beta }}_{n}$ is consistent with ${\hat{\beta }}_{N}$, i.e.,*

$$∥{\hat{\beta }}_{n}-{\hat{\beta }}_{N}∥=o_{P|F_{N}}\left(1\right)$$

*where $o_{P|F_{N}}\left(1\right)$ means o(1) conditioning on $F_{N}$ in probability.*

**Theorem** **3.** *Under the conditions in Theorem 2, as N→∞ and n→∞, conditional on $F_{N}$ in probability,*

$$\sqrt{n}\left({\hat{\beta }}_{n}-{\hat{\beta }}_{N}\right)\to N\left(0,V_{s}\right),$$

*in distribution, where*

$$\begin{array}{cc} & V_{s}=\Sigma_{N}^{-1}V_{N}\Sigma_{N}^{-1},\\ & \Sigma_{N}=\sum_{i\in U}a_{i}\left[Y_{i}-\dot{b}\left(\psi \left({\hat{\beta }}_{N}^{T}X_{i}\right)\right)\right]\ddot{\psi }\left({\hat{\beta }}_{N}^{T}X_{i}\right)X_{i}X_{i}^{T}-\sum_{i\in U}a_{i}\ddot{b}\left(\psi \left({\hat{\beta }}_{N}^{T}X_{i}\right)\right)\dot{\psi }{\left({\hat{\beta }}_{N}^{T}X_{i}\right)}^{2}X_{i}X_{i}^{T},\\ & V_{N}=\sum_{i\in U}\frac{a_{i}}{\pi_{i}}{\left[Y_{i}-\dot{b}\left(\psi \left({\hat{\beta }}_{N}^{T}X_{i}\right)\right)\right]}^{2}\dot{\psi }{\left({\hat{\beta }}_{N}^{T}X_{i}\right)}^{2}X_{i}X_{i}^{T}. \end{array}$$

In this part, we complete the asymptotic properties without the moment condition of the covariates ${\left\{X_{i}\right\}}_{i=1}^{N}$ which is used in [9], and that means $X_{i}$’s are unbounded. Here we only provide the theoretical asymptotic results. Furthermore, the subsampling probability can be derived by A-optimal criterion like [10].

### 3.3. Unconditional Asymptotic Properties of Subsampled GLMs with Unbounded Covariates

In real engineering, the measurement of some response variable data is very expensive, such as superconductor data, deep space exploration data, etc. The accuracy of estimating the target parameters under measurement constraints of responses is a very important issue. Ref. [19] completed the unconditional asymptotic properties of parameter estimation in bounded GLMs with canonical link. But the unbounded GLMs with nonnatural link situation has not been discussed yet.

In this section, we continue to use the notations of Section 3.2. Through the theory of empirical process [11], we obtain the unconditional consistency of ${\hat{\beta }}_{n}$ in the following theorem.

**Theorem** **4.** *(Unconditional subsampled consistency) Assume the conditions:*

*(B.1)*  *$\lambda_{\min}\left(EXX^{T}\right)>0$ where X is the unbounded covariate of GLMs.*
*(B.2)*  *For $\forall u_{1},u_{2}\in \left[0,1\right]$,*
$$\underset{\beta \in \Theta \backslash \{\beta_{0}\}}{\inf}\frac{E\{\ddot{b}\left({\tilde{\psi }}_{u_{1}}\right)\dot{\psi }{\left[\left(1-u_{2}\right)\left(\beta_{0}^{T}X\right)+u_{2}\left(\beta^{T}X\right)\right]}^{2}{\left(\beta^{T}X-\beta_{0}^{T}X\right)}^{2}\}}{E{\left(\beta^{T}X-\beta_{0}^{T}X\right)}^{2}}\geq C_{1}>0,$$
  *where ${\tilde{\psi }}_{u_{1}}=\left(1-u_{1}\right)\psi \left(\beta_{0}^{T}X\right)+u_{1}\psi \left(\beta^{T}X\right)$ and $\ddot{b}\left(\cdot \right)$ is the second derivative with respect to β.*
*(B.3)* 
$$E_{\beta_{0}}\underset{\beta \in \Theta }{\sup}[|Y-\dot{b}\left(\psi \left(\beta^{T}X\right)\right){\left|\cdot ||X|\right|}^{2}]<\infty ,$$
  *where $\dot{b}\left(\cdot \right)$ is the first derivative with respect to β.*
*(B.4)*  *ψ(·) in (3) is twice continuously differentiable and its every derivative has a positive minimum.*
*(B.5)*  *b(·) in (3) is twice continuously differentiable and its every derivative has a positive minimum.*

*Then $∥{\hat{\beta }}_{n}-\beta_{0}∥=o_{P}\left(1\right)$.*

Theorem 4 directly obtains the unconditional consistency of the subsampling estimator with respect to the true parameters under the unbounded assumption.

To prove the asymptotic normality of ${\hat{\beta }}_{n}$ with respect to $\beta_{0}$, we briefly review the subsampled score function in Section 3.2

$$s_{n}\left(\beta \right)=\sum_{i\in S}\frac{1}{\pi_{i}^{*}}\left[Y_{i}^{*}-\mu \left(\psi \left(\beta^{T}X_{i}^{*}\right)\right)\right]\dot{\psi }\left(\beta^{T}X_{i}^{*}\right)X_{i}^{*}:=\sum_{i\in S}\frac{1}{\pi_{i}^{*}}\phi_{\beta }\left(X_{i}^{*},Y_{i}^{*}\right).$$

Next we will apply a multivariate martingale central limit theorem (Lemma 4 in [19]), which is the extension of Theorem A.1 in [20], to show the asymptotic normality of ${\hat{\beta }}_{n}$. Let ${\left\{F_{N,i}\right\}}_{i=1}^{n}$ be a filtration adaptive to the sampling: $F_{N,0}=\sigma \left(X_{1}^{N},Y_{1}^{N}\right);F_{N,1}=\sigma \left(X_{1}^{N},Y_{1}^{N}\right)\vee \sigma \left({*}_{1}\right);\ldots ;F_{N,i}=\sigma \left(X_{1}^{N},Y_{1}^{N}\right)\vee \sigma \left({*}_{1}\right)\vee \ldots \vee \sigma \left({*}_{i}\right);\ldots$, where $\sigma ({*}_{i})$ is the σ-algebra generated by *i*th sampling step. The subsample of size *n* is assumed to increase with *N*. By the filtration, we define the martingale

$$\bar{M}:=\sum_{i=1}^{n}{\bar{M}}_{i}:=\sum_{i=1}^{n}\left[\frac{1}{\pi_{i}^{*}}\phi_{\beta }\left(X_{i}^{*},Y_{i}^{*}\right)-\sum_{j=1}^{N}\phi_{\beta }\left(X_{j},Y_{j}\right)\right],$$

where ${\left\{{\bar{M}}_{i}\right\}}_{i=1}^{n}$ is a martingale difference sequence adapted to ${\left\{F_{N,i}\right\}}_{i=1}^{n}$. In addition, define $Q:=n\sum_{j=1}^{N}\phi_{\beta }\left(X_{j},Y_{j}\right)$; $T:=s_{n}\left(\beta \right)=\bar{M}+Q$; $\xi_{Ni}:={\mathrm{Var}}^{-1/2}\left(T\right){\bar{M}}_{i}$ and $B_{N}:={\mathrm{Var}}^{-1/2}\left(T\right)\mathrm{Var}\left(\bar{M}\right){\mathrm{Var}}^{-1/2}\left(T\right)$, where matrix $A^{1/2}$ is the symmetric square root of A, i.e., $A={\left(A^{1/2}\right)}^{2}$, and $A^{-1/2}={\left(A^{1/2}\right)}^{-1}={\left(A^{-1}\right)}^{1/2}$. $B_{N}$ is the variance of ${\mathrm{Var}}^{-1/2}\left(T\right)\bar{M}$.

The following theorem shows the asymptotic normality of the estimator ${\hat{\beta }}_{n}$.

**Theorem** **5.** *Assume the conditions,*

*(C.1)* 
$$\Phi =E\left(\nabla s_{n}\left(\beta \right)\right)=E\left[-\sum_{i\in S}\frac{1}{\pi_{i}^{*}}\dot{\mu }\left(\psi \left(\beta^{T}X_{i}^{*}\right)\right){\left[\dot{\psi }\left(\beta^{T}X_{i}^{*}\right)\right]}^{2}X_{i}^{*}X_{i}^{*T}\right]$$
  *is finite and nonsingular.*
*(C.2)*  *$E\left\{{\left[\sum_{i\in U}\frac{a_{i}}{\pi_{i}}\dot{\mu }\left(\psi \left(\beta^{T}X_{i}\right)\right){\left[\dot{\psi }\left(\beta^{T}X_{i}\right)\right]}^{2}X_{ik}X_{ij}\right]}^{2}\right\}=o_{P}\left(1\right)$, for 1≤k,j≤p,* *where $X_{ik}$ means k-th element of vector $X_{i}$ and $X_{ij}$ means j-th element of vector $X_{i}$.*
*(C.3)*  *ψ(x) is three-times continuously differentiable for every x with its domain.*
*(C.4)*  *For any i∈S, $\left|\right|{\ddot{\phi }}_{\beta }\left(X_{i}^{*},Y_{i}^{*}\right)\left|\right|<\infty$.*
*(C.5)*  *$\min_{i=1,...,N}\left(N\pi_{i}\right)=\max_{i=1,...,N}\left(N\pi_{i}\right)=O\left(1\right)$ and n/N=o(1).*
*(C.6)*  *$\lim_{N\to \infty }\sum_{i=1}^{n}E[||\xi_{Ni}{\left|\right|}^{4}]=0$,*
*(C.7)* 
$\lim_{N\to \infty }E\left[{\left|\left|\sum_{i=1}^{n}E\left[\xi_{Ni}\xi_{Ni}^{T}|F_{N,i-1}\right]-B_{N}\right|\right|}^{2}\right]=0.$

*Then*

$$\mathrm{Var}{\left(T\right)}^{-1/2}\Phi \left({\hat{\beta }}_{n}-\beta_{0}\right)\overset{d}{\to }N\left(0,I_{p}\right).$$

Here, we establish the unconditional asymptotic properties of subsampling estimator for unbounded GLMs. The condition n/N=o(1) ensures that small-scale subsamples also have expected performance, which greatly release the computational cost. We also present the theoretical asymptotic results, which leads to the subsampling probability using the A-optimal criterion in [10].

## 4. Conclusions and Future Work

In this paper, we derive the asymptotic normality of the subsampling M-estimator by Fisher information. In the unbounded GLMs with nonnatural link function, we separately obtain the conditional and unconditional asymptotic properties of subsampling estimator.

For future study, it is meaningful to apply the sub-Weibull concentration inequalities in [21] to make nonasymptotic inference. The importance sampling is not ideal, since it tends to assign high sampling probability to the observed samples. Hence, effective subsampling methods are considered for GLMs, such as Markov subsampling in [22]. Moreover, high-dimensional methods in [23,24] for subsampling need further studies. 

## Data Availability

Not applicable.

## Appendix A. Technical Details

**Lemma** **A1** (Theorem 4.17 in [16])**.** *Let $X_{1},\ldots ,X_{N}$ be i.i.d. from a p.d.f. $f_{\beta }$ w.r.t. a σ-finite measure ν on $\left(R,B_{R}\right)$, where β∈Θ and *Θ* is an open set in $R^{p}$. Suppose that for every x in the range of $X_{1}$, $f_{\beta }\left(x\right)$ is twice continuously differentiable on β and satisfies*

(D.1) $$\frac{\partial }{\partial \beta }\int \psi_{\beta }\left(x\right)d\nu =\int \frac{\partial }{\partial \beta }\psi_{\beta }\left(x\right)d\nu$$
  *for $\psi_{\beta }\left(x\right)=f_{\beta }\left(x\right)$ and $\psi_{\beta }\left(x\right)=\frac{\partial f_{\beta }\left(x\right)}{\partial \beta }$.*
(D.2) *The Fisher information matrix*
  $$I_{1}\left(\beta \right)=E\left\{\frac{\partial }{\partial \beta }\log f_{\beta }\left(X_{1}\right){\left[\frac{\partial }{\partial \beta }\log f_{\beta }\left(X_{1}\right)\right]}^{T}\right\}$$
  *is positive definite.*
(D.3) *For any given β∈Θ, there exists a positive number $C_{\beta }$ and a positive function $h_{\beta }$ such that $E[h_{\beta }\left(X_{1}\right)]<\infty$ and*
  $$\underset{\gamma :∥\gamma -\beta ∥<C_{\beta }}{\sup}∥\frac{\partial^{2}\log f_{\gamma }\left(x\right)}{\partial \gamma \partial \gamma^{T}}∥\leq h_{\beta }\left(x\right)$$
  *for all x in the range of $X_{1}$, where ||·|| is Euclidean norm and $∥A∥=\sqrt{\mathrm{tr}(A^{T}A)}$ for any matrix A. Then there exist a sequence of estimators ${\hat{\beta }}_{N}$ (based on $\left\{X_{i},i\in U\right\}$) such that*
  (A1) $$P\left(s_{a}\left({\hat{\beta }}_{N}\right)=0\right)\to 1\;\mathrm{and}\;{\hat{\beta }}_{N}\overset{P}{\to }\beta_{0},$$
  *where $s_{a}\left(\gamma \right)=\frac{\partial \log{\tilde{L}}_{N}\left(\gamma \right)}{\partial \gamma }$ and ${\tilde{L}}_{N}\left(\gamma \right)$ is the likelihood function of full data and $\beta_{0}$ is the real parameter. Meanwhile, there exist a sequence of estimators ${\hat{\beta }}_{n}$ (based on $\left\{X_{i},i\in S\right\}$) such that*
  (A2) $$P\left(s_{s}\left({\hat{\beta }}_{n}\right)=0\right)\to 1\;\mathrm{and}\;{\hat{\beta }}_{n}\overset{P}{\to }\beta_{0},$$
  *where $s_{s}\left(\gamma \right)=\frac{\partial \log{\tilde{L}}_{n}\left(\gamma \right)}{\partial \gamma }$ and ${\tilde{L}}_{n}\left(\gamma \right)$ is the likelihood function of subsampled data and $\beta_{0}$ is the real parameter.*

Let $a_{i}$ be the number of *i*-th subsampled data such that $\sum_{i\in U}a_{i}=n$.

**Lemma** **A2.**
*$E\left[L_{n}\left(\beta \right)\right|F_{N}]=L_{N}\left(\beta \right)$.*

**Proof.** From the definition of $a_{i}$, one has

$$\begin{array}{cc} E[L_{n}\left(\beta \right)|F_{N}] & =E\left[\frac{1}{Nn}\sum_{i\in S}\frac{1}{\pi_{i}^{*}}l\left(\beta ;Z_{i}^{*}\right)|F_{N}\right]\\ & =E\left[\frac{1}{Nn}\sum_{i\in U}\frac{1}{\pi_{i}}l\left(\beta ;Z_{i}\right)a_{i}|F_{N}\right]\\ & =\frac{1}{Nn}\sum_{i\in U}a_{i}E\left[\frac{1}{\pi_{i}}l\left(\beta ;Z_{i}\right)|F_{N}\right]\\ & =\frac{1}{Nn}\sum_{i\in U}a_{i}\frac{\sum_{i\in U}\frac{1}{\pi_{i}}l\left(\beta ;Z_{i}\right)\pi_{i}}{\sum_{i\in U}\pi_{i}}\\ & =\frac{1}{Nn}\sum_{i\in U}a_{i}\sum_{i\in U}l\left(\beta ;Z_{i}\right)\\ & =\frac{1}{Nn}n\sum_{i\in U}l\left(\beta ;Z_{i}\right)\\ & =\frac{1}{N}\sum_{i\in U}l\left(\beta ;Z_{i}\right)\\ & =L_{N}\left(\beta \right). \end{array}$$

□

**Proposition** **A1.**
*Under the conditions of Lemma A1 and*

$$\min_{i}\left(N\pi_{i}\right)=\max_{i}\left(N\pi_{i}\right)=O\left(1\right)\left(i=1,\ldots ,N\right).$$

*Assume that ${\hat{\beta }}_{N}$ based on $\left\{X_{i},i\in U\right\}$ is an estimator of β, and ${\hat{\beta }}_{n}$ based on $\left\{X_{i},i\in S\right\}$ is also an estimator of β, then*

(A3)
$${\hat{\beta }}_{n}-{\hat{\beta }}_{N}=-\Sigma_{N}^{-1}\nabla L_{n}\left({\hat{\beta }}_{N}\right)+o_{P|F_{N}}\left(1\right).$$

**Proof.** Taking Taylor series expansion of $\nabla L_{n}\left({\hat{\beta }}_{n}\right)$ around ${\hat{\beta }}_{N}$, we have

(A4) $$\begin{array}{cc} 0= & \nabla L_{n}\left({\hat{\beta }}_{n}\right)\\ = & \nabla L_{n}\left({\hat{\beta }}_{N}\right)+\nabla^{2}L_{n}\left({\hat{\beta }}_{N}\right)\left({\hat{\beta }}_{n}-{\hat{\beta }}_{N}\right)+o\left(∥{\hat{\beta }}_{n}-{\hat{\beta }}_{N}∥\right)\\ = & \nabla L_{n}\left({\hat{\beta }}_{N}\right)+\nabla^{2}L_{n}\left({\hat{\beta }}_{N}\right)\left({\hat{\beta }}_{n}-{\hat{\beta }}_{N}\right)+\nabla^{2}L_{N}\left({\hat{\beta }}_{N}\right)\left({\hat{\beta }}_{n}-{\hat{\beta }}_{N}\right)\\ \; & -\nabla^{2}L_{N}\left({\hat{\beta }}_{N}\right)\left({\hat{\beta }}_{n}-{\hat{\beta }}_{N}\right)+o\left(∥{\hat{\beta }}_{n}-{\hat{\beta }}_{N}∥\right)\\ = & \nabla L_{n}\left({\hat{\beta }}_{N}\right)+\nabla^{2}L_{N}\left({\hat{\beta }}_{N}\right)\left({\hat{\beta }}_{n}-{\hat{\beta }}_{N}\right)+\left(\nabla^{2}L_{n}\left({\hat{\beta }}_{N}\right)-\nabla^{2}L_{N}\left({\hat{\beta }}_{N}\right)\right)\left({\hat{\beta }}_{n}-{\hat{\beta }}_{N}\right)\\ \; & +o(∥{\hat{\beta }}_{n}-{\hat{\beta }}_{N}∥). \end{array}$$

From the definition of $a_{i}$, one has

(A5) $$\begin{array}{cc} \left(\nabla^{2}L_{n}\left({\hat{\beta }}_{N}\right)-\nabla^{2}L_{N}\left({\hat{\beta }}_{N}\right)\right)\left({\hat{\beta }}_{n}-{\hat{\beta }}_{N}\right)= & \left(\frac{1}{Nn}\sum_{i\in S}\frac{1}{\pi_{i}^{*}}\nabla^{2}l\left({\hat{\beta }}_{N};Z_{i}^{*}\right)-\frac{1}{N}\sum_{i\in U}\nabla^{2}l\left({\hat{\beta }}_{N};Z_{i}\right)\right)\\ \; & \cdot ({\hat{\beta }}_{n}-{\hat{\beta }}_{N})\\ = & \left(\sum_{i\in U}\frac{1}{Nn\pi_{i}}\nabla^{2}l\left({\hat{\beta }}_{N};Z_{i}\right)a_{i}-\frac{1}{N}\sum_{i\in U}\nabla^{2}l\left({\hat{\beta }}_{N};Z_{i}\right)\right)\\ \; & \cdot ({\hat{\beta }}_{n}-{\hat{\beta }}_{N})\\ = & \left(\sum_{i\in U}\frac{a_{i}-n\pi_{i}}{Nn\pi_{i}}\nabla^{2}l\left({\hat{\beta }}_{N};Z_{i}\right)\right)\left({\hat{\beta }}_{n}-{\hat{\beta }}_{N}\right)\\ \leq & \left(\sum_{i\in U}\frac{a_{i}}{Nn\pi_{i}}\nabla^{2}l\left({\hat{\beta }}_{N};Z_{i}\right)\right)\left({\hat{\beta }}_{n}-{\hat{\beta }}_{N}\right)\\ = & o_{P|F_{N}}\left(1\right). \end{array}$$

Combine Equations (A1), (A2) and (A5) into Equation (A4), one has

$$0=\nabla L_{n}\left({\hat{\beta }}_{N}\right)+\nabla^{2}L_{N}\left({\hat{\beta }}_{N}\right)\left({\hat{\beta }}_{n}-{\hat{\beta }}_{N}\right)+o_{P|F_{N}}\left(1\right).$$

This can be transformed to

(A6) $${\hat{\beta }}_{n}-{\hat{\beta }}_{N}=-\Sigma_{N}^{-1}\nabla L_{n}\left({\hat{\beta }}_{N}\right)+o_{P|F_{N}}\left(1\right).$$

The proposition is proved. □

**Remark** **A1.**
*The last equation in the proof ensures that ${\hat{\beta }}_{n}-{\hat{\beta }}_{N}+\Sigma_{N}^{-1}\nabla L_{n}\left({\hat{\beta }}_{N}\right)$ is a higher-order infinitesimal with respect to 1, which is true according to conditional probability with $F_{N}$. $o_{P|F_{N}}\left(1\right)$ in Equation (A6) is denoted as the higher order infinitesimal of 1 according to conditional probability with $F_{N}$.*

**Proof of** **Theorem 1.** For every constant $\hat{\gamma }>0$, one has

$$\begin{array}{cc} \sum_{j\in S}E\left\{{∥n^{-\frac{1}{2}}\zeta_{j}^{*}∥}^{2}I\left(∥\zeta_{j}^{*}∥>n^{\frac{1}{2}}\hat{\gamma }\right)|F_{N}\right\} & \leq \sum_{j\in S}E\left\{\frac{{∥n^{-\frac{1}{2}}∥}^{2}{∥\zeta_{j}^{*}∥}^{4}}{n{\hat{\gamma }}^{2}}I\left(∥\zeta_{j}^{*}∥>n^{\frac{1}{2}}\hat{\gamma }\right)|F_{N}\right\}\\ & =\frac{1}{n^{2}{\hat{\gamma }}^{2}}\sum_{j\in S}E\left\{{∥\zeta_{j}^{*}∥}^{4}I\left(∥\zeta_{j}^{*}∥>n^{\frac{1}{2}}\hat{\gamma }\right)|F_{N}\right\}\\ & \leq \frac{1}{n^{2}{\hat{\gamma }}^{2}}\sum_{j\in S}E\left\{{∥\zeta_{j}^{*}∥}^{4}|F_{N}\right\}\\ & =\frac{1}{n^{2}{\hat{\gamma }}^{2}}\sum_{i\in U}a_{i}E\left\{{∥\zeta_{i}∥}^{4}|F_{N}\right\}\\ & =\frac{1}{n^{2}{\hat{\gamma }}^{2}}n\frac{\sum_{i\in U}{∥\zeta_{i}∥}^{4}\pi_{i}}{\sum_{i\in U}\pi_{i}}\\ & =\frac{1}{n{\hat{\gamma }}^{2}}\sum_{i\in U}{∥\frac{1}{N\pi_{i}}\Sigma_{N}^{-1}\nabla l\left({\hat{\beta }}_{N};Z_{i}\right)∥}^{4}\pi_{i}\\ & =\frac{1}{n{\hat{\gamma }}^{2}}\frac{1}{N^{4}}\sum_{i\in U}\frac{1}{\pi_{i}^{3}}{∥\Sigma_{N}^{-1}\nabla l\left({\hat{\beta }}_{N};Z_{i}\right)∥}^{4}\\ & \leq \frac{1}{n{\hat{\gamma }}^{2}}\frac{1}{N^{4}}\sum_{i\in U}\frac{1}{\pi_{i}^{3}}\frac{1}{\lambda^{4}}{∥\nabla l({\hat{\beta }}_{N};Z_{i})∥}^{4}\\ & =\frac{1}{n{\hat{\gamma }}^{2}}\frac{1}{\lambda^{4}}O\left(1\right)\\ & =o(1). \end{array}$$

Furthermore,

$$\begin{array}{cc} \sum_{j\in S}\mathrm{Cov}\left(n^{-\frac{1}{2}}\zeta_{j}^{*}|F_{N}\right) & =\sum_{j\in S}E\left\{\left[n^{-\frac{1}{2}}\zeta_{j}^{*}-E\left(n^{-\frac{1}{2}}\zeta_{j}^{*}|F_{N}\right)\right]{\left[n^{-\frac{1}{2}}\zeta_{j}^{*}-E\left(n^{-\frac{1}{2}}\zeta_{j}^{*}|F_{N}\right)\right]}^{T}|F_{N}\right\}\\ & =\sum_{j\in S}E\left[\left(n^{-\frac{1}{2}}\zeta_{j}^{*}\right){\left(n^{-\frac{1}{2}}\zeta_{j}^{*}\right)}^{T}|F_{N}\right]\\ & =\frac{1}{n}\sum_{j\in S}E\left(\zeta_{j}^{*}\zeta_{j}^{*T}|F_{N}\right)\\ & =\frac{1}{n}nE\left(\zeta \zeta^{T}|F_{N}\right)\\ & =\mathrm{Var}(\zeta |F_{N}). \end{array}$$

Then, by the Lindeberg-Feller central limit theorem (Proposition 2.27 of [11]), conditional on $F_{N}$,

$$\begin{array}{c} \sum_{j\in S}n^{-\frac{1}{2}}\zeta_{j}^{*}\overset{d}{\to }N\left(0,\mathrm{Var}\left(\zeta |F_{N}\right)\right). \end{array}$$

Therefore, combining the above and Proposition A1, Equation (5) holds. Thus, the proof is completed. □

**Proof of** **Theorem 2.** Next, one needs to show convexity (i.e., uniqueness and maximum value) due to the existence of the estimators from [25]. Let

$$\begin{array}{cc} I_{1}\left(\beta \right)= & E(\nabla s_{n}\left(\beta \right))\\ = & E\left(\frac{\partial \left(\sum_{i\in S}\frac{1}{\pi_{i}^{*}}\left[Y_{i}^{*}-\mu \left(\psi \left(\beta^{T}X_{i}^{*}\right)\right)\right]\dot{\psi }\left(\beta^{T}X_{i}^{*}\right)X_{i}^{*}\right)}{\partial \beta }\right)\\ = & E(-\sum_{i\in S}\frac{1}{\pi_{i}^{*}}\dot{\mu }\left(\psi \left(\beta^{T}X_{i}^{*}\right)\right){\left[\dot{\psi }\left(\beta^{T}X_{i}^{*}\right)\right]}^{2}X_{i}^{*}X_{i}^{*T}\\ & +\sum_{i\in S}\frac{1}{\pi_{i}^{*}}\left[Y_{i}^{*}-\mu \left(\psi \left(\beta^{T}X_{i}^{*}\right)\right)\right]\ddot{\psi }\left(\beta^{T}X_{i}^{*}\right)X_{i}^{*}X_{i}^{*T})\\ = & -\sum_{i\in S}\frac{1}{\pi_{i}^{*}}\dot{\mu }\left(\psi \left(\beta^{T}X_{i}^{*}\right)\right){\left[\dot{\psi }\left(\beta^{T}X_{i}^{*}\right)\right]}^{2}X_{i}^{*}X_{i}^{*T}, \end{array}$$

where

$$s_{n}\left(\beta \right)=\sum_{i\in S}\frac{1}{\pi_{i}^{*}}\left[Y_{i}^{*}-\mu \left(\psi \left(\beta^{T}X_{i}^{*}\right)\right)\right]\dot{\psi }\left(\beta^{T}X_{i}^{*}\right)X_{i}^{*}.$$

From [16] in Theorem 4.17, one needs to show

$$\max_{\gamma \in G(C_{0})}∥{\left[I_{1}\left({\hat{\beta }}_{N}\right)\right]}^{-1/2}\nabla s_{n}\left(\gamma \right){\left[I_{1}\left({\hat{\beta }}_{N}\right)\right]}^{-1/2}+I_{p}∥\overset{P|F_{N}}{\to }0,$$

where $G\left(C_{0}\right)=\left\{\gamma :∥{\left[I_{1}\left({\hat{\beta }}_{N}\right)\right]}^{1/2}\left(\gamma -{\hat{\beta }}_{N}\right)∥\leq C_{0}\right\}$ and $I_{p}=\mathrm{diag}\left(1,1,\ldots ,1\right)$ is a *p*-dimensional identity matrix. Let

(A7) $$M_{n}\left(\gamma \right)=\sum_{i\in S}\frac{1}{\pi_{i}^{*}}{\left[\dot{\psi }\left(\gamma^{T}X_{i}^{*}\right)\right]}^{2}\ddot{b}\left(\psi \left(\gamma^{T}X_{i}^{*}\right)\right)X_{i}^{*}X_{i}^{*T}$$

and

(A8) $$R_{n}\left(\gamma \right)=\sum_{i\in S}\frac{1}{\pi_{i}^{*}}\left[Y_{i}^{*}-\mu \left(\psi \left(\gamma^{T}X_{i}^{*}\right)\right)\right]\ddot{\psi }\left(\gamma^{T}X_{i}^{*}\right)X_{i}^{*}X_{i}^{*T}.$$

Then

(A9) $$\nabla s_{n}\left(\gamma \right)=R_{n}\left(\gamma \right)-M_{n}\left(\gamma \right)$$

and

(A10) $$I_{1}\left(\gamma \right)=-E\left(\nabla s_{n}\left(\gamma \right)\right)=M_{n}\left(\gamma \right)$$

Thus, one only needs to prove

(A11) $$\max_{\gamma \in G(C_{0})}∥{\left[M_{n}\left({\hat{\beta }}_{N}\right)\right]}^{-1/2}\left[M_{n}\left(\gamma \right)-M_{n}\left({\hat{\beta }}_{N}\right)\right]{\left[M_{n}\left({\hat{\beta }}_{N}\right)\right]}^{-1/2}∥\overset{P|F_{N}}{\to }0,$$

and

(A12) $$\max_{\gamma \in G(C_{0})}∥{\left[M_{n}\left({\hat{\beta }}_{N}\right)\right]}^{-1/2}R_{n}\left(\gamma \right){\left[M_{n}\left({\hat{\beta }}_{N}\right)\right]}^{-1/2}∥\overset{P|F_{N}}{\to }0$$

for any $C_{0}>0$. From the definition of $M_{n}\left(\gamma \right)$, and the property of trace in P288 of [16], the left-hand side of Equation (A11) can be bounded by

$$\sqrt{p}\max_{\gamma \in G(C_{0}),i\in S}\left|1-\varphi \left(\gamma^{T}X_{i}^{*}\right)/\varphi \left({\hat{\beta }}_{N}^{T}X_{i}^{*}\right)\right|.$$

From condition (A.4), one needs to prove $\left|\gamma^{T}X_{i}^{*}-{\hat{\beta }}_{N}^{T}X_{i}^{*}\right|$ converges to 0 so that Equation (A11) holds, and one has

γTXi∗−β^NTXi∗2=(γT−β^NT)I1(β^N)1/2I1(β^N)−1/2Xi∗2≤I1(β^N)1/2(γ−β^N)2I1(β^N)−1/2Xi∗2≤C02maxi∈SXi∗TI1(β^N)−1Xi∗=C02maxi∈SXi∗TMn(β^N)−1Xi∗=C02maxi∈SXi∗T∑i∈S1πi∗[ψ˙(^}βNTXi∗)]2b¨(ψ(^}βNTXi∗))Xi∗Xi∗T−1Xi∗=C02maxi∈SXi∗T∑i∈S1πi∗φ(^}βNTXi∗)Xi∗Xi∗T−1Xi∗=C02maxi∈SXi∗T∑i∈SN1Nπi∗φ(^}βNTXi∗)Xi∗Xi∗T−1Xi∗≤C02Nmini∈S1Nπi∗infi∈Sφβ^NTXi∗−1maxi∈SXi∗T∑i∈SXi∗Xi∗T−1Xi∗=C02Nmini∈S1Nπi∗infi∈Sφβ^NTXi∗−1maxi∈SXi∗TX∗X∗T−1Xi∗→P|FN0.

Hence Equation (A11) holds. Let $e_{i}^{*}=Y_{i}^{*}-\mu \left(\psi \left({\hat{\beta }}_{N}^{T}X_{i}^{*}\right)\right)$, and

$$\begin{array}{cc} & U_{n}\left(\gamma \right)=\sum_{i\in S}\frac{1}{\pi_{i}^{*}}\left[\mu \left(\psi \left({\hat{\beta }}_{N}^{T}X_{i}^{*}\right)\right)-\mu \left(\psi \left(\gamma^{T}X_{i}^{*}\right)\right)\right]\ddot{\psi }\left(\gamma^{T}X_{i}^{*}\right)X_{i}^{*}X_{i}^{*T},\\ & V_{n}\left(\gamma \right)=\sum_{i\in S}\frac{e_{i}^{*}}{\pi_{i}^{*}}\left[\ddot{\psi }\left(\gamma^{T}X_{i}^{*}\right)-\ddot{\psi }\left({\hat{\beta }}_{N}^{T}X_{i}^{*}\right)\right]X_{i}^{*}X_{i}^{*T},\\ & W_{n}\left({\hat{\beta }}_{N}\right)=\sum_{i\in S}\frac{e_{i}^{*}}{\pi_{i}^{*}}\ddot{\psi }\left({\hat{\beta }}_{N}^{T}X_{i}^{*}\right)X_{i}^{*}X_{i}^{*T}. \end{array}$$

Then $R_{n}\left(\gamma \right)=U_{n}\left(\gamma \right)+V_{n}\left(\gamma \right)+W_{n}\left({\hat{\beta }}_{N}\right)$. In the same way as proving Equation (A11), we have

$$\max_{\gamma \in G(C_{0})}∥{\left[M_{n}\left({\hat{\beta }}_{N}\right)\right]}^{-1/2}U_{n}\left(\gamma \right){\left[M_{n}\left({\hat{\beta }}_{N}\right)\right]}^{-1/2}∥\overset{P|F_{N}}{\to }0.$$

Note that $∥{\left[M_{n}\left({\hat{\beta }}_{N}\right)\right]}^{-1/2}V_{n}\left(\gamma \right){\left[M_{n}\left({\hat{\beta }}_{N}\right)\right]}^{-1/2}∥$ is bounded by the product of

(A13) $$∥{\left[M_{n}\left({\hat{\beta }}_{N}\right)\right]}^{-\frac{1}{2}}\sum_{i\in S}\frac{e_{i}^{*}}{\pi_{i}^{*}}X_{i}^{*}X_{i}^{*T}{\left[M_{n}\left({\hat{\beta }}_{N}\right)\right]}^{-\frac{1}{2}}∥$$

and

(A14) $$\max_{\gamma \in G(C_{0}),i\in S}\left|\ddot{\psi }\left(\gamma^{T}X_{i}^{*}\right)-\ddot{\psi }\left({\hat{\beta }}_{N}^{T}X_{i}^{*}\right)\right|.$$

Equation (A13) can be bounded as

$$\begin{array}{cc} & ∥{\left[M_{n}\left({\hat{\beta }}_{N}\right)\right]}^{-\frac{1}{2}}\sum_{i\in S}\frac{e_{i}^{*}}{\pi_{i}^{*}}X_{i}^{*}X_{i}^{*T}{\left[M_{n}\left({\hat{\beta }}_{N}\right)\right]}^{-\frac{1}{2}}∥\\ = & ∥{\left[I_{1}\left({\hat{\beta }}_{N}\right)\right]}^{-\frac{1}{2}}\sum_{i\in S}\frac{e_{i}^{*}}{\pi_{i}^{*}}X_{i}^{*}X_{i}^{*T}{\left[I_{1}\left({\hat{\beta }}_{N}\right)\right]}^{-\frac{1}{2}}∥\\ \leq & \left|\sum_{i\in S}\frac{e_{i}^{*}}{\pi_{i}^{*}}\right|{∥{\left[I_{1}\left({\hat{\beta }}_{N}\right)\right]}^{-\frac{1}{2}}X_{i}^{*}∥}^{2}\\ \leq & \left|\sum_{i\in S}\frac{e_{i}^{*}}{\pi_{i}^{*}}\right|{\left[N\min_{i\in S}\frac{1}{N\pi_{i}^{*}}\underset{i\in S}{\inf}\varphi \left({\hat{\beta }}_{N}^{T}X_{i}^{*}\right)\right]}^{-1}\max_{i\in S}X_{i}^{*T}{\left(X^{*}X^{*T}\right)}^{-1}X_{i}^{*}\\ \leq & \left|\sum_{i\in S}e_{i}^{*}\right|\left|\max_{i\in S}\frac{1}{N\pi_{i}^{*}}\right|{\left[\min_{i\in S}\frac{1}{N\pi_{i}^{*}}\underset{i\in S}{\inf}\varphi \left({\hat{\beta }}_{N}^{T}X_{i}^{*}\right)\right]}^{-1}\max_{i\in S}X_{i}^{*T}{\left(X^{*}X^{*T}\right)}^{-1}X_{i}^{*}\\ \leq & \sum_{i\in U}\left|Y_{i}-\mu \left(\psi \left({\hat{\beta }}_{N}^{T}X_{i}\right)\right)\right|\left|\max_{i\in S}\frac{1}{N\pi_{i}^{*}}\right|{\left[\min_{i\in S}\frac{1}{N\pi_{i}^{*}}\underset{i\in S}{\inf}\varphi \left({\hat{\beta }}_{N}^{T}X_{i}^{*}\right)\right]}^{-1}\max_{i\in S}X_{i}^{*T}{\left(X^{*}X^{*T}\right)}^{-1}X_{i}^{*}\\ \leq & \left[\sum_{i\in U}\underset{\beta \in \Theta }{\sup}\left|Y_{i}-\mu \left(\psi \left(\beta^{T}X_{i}\right)\right)\right|\right]\left|\max_{i\in S}\frac{1}{N\pi_{i}^{*}}\right|{\left[\min_{i\in S}\frac{1}{N\pi_{i}^{*}}\underset{i\in S}{\inf}\varphi \left({\hat{\beta }}_{N}^{T}X_{i}^{*}\right)\right]}^{-1}\\ & \cdot \max_{i\in S}X_{i}^{*T}{\left(X^{*}X^{*T}\right)}^{-1}X_{i}^{*}\\ = & \frac{1}{n}\sum_{i\in S}E\underset{\beta \in \Theta }{\sup}\left[\frac{1}{\pi_{i}^{*}}\left|Y_{i}^{*}-\mu \left(\psi \left(\beta^{T}X_{i}^{*}\right)\right)\right||F_{N}\right]\left|\max_{i\in S}\frac{1}{N\pi_{i}^{*}}\right|{\left[\min_{i\in S}\frac{1}{N\pi_{i}^{*}}\underset{i\in S}{\inf}\varphi \left({\hat{\beta }}_{N}^{T}X_{i}^{*}\right)\right]}^{-1}\\ \cdot & \max_{i\in S}X_{i}^{*T}{\left(X^{*}X^{*T}\right)}^{-1}X_{i}^{*}\\ = & O_{P|F_{N}}\left(1/n\right), \end{array}$$

where the penultimate equal sign applies the Lemma A2 with

$$l\left(\beta \right)=\underset{\beta \in \Theta }{\sup}\left|Y_{i}-\mu \left(\psi \left(\beta^{T}X_{i}\right)\right)\right|.$$

Equation (A14) can be bounded as

$$\max_{\gamma \in G(C_{0}),i\in S}\left|\ddot{\psi }\left(\gamma^{T}X_{i}^{*}\right)-\ddot{\psi }\left({\hat{\beta }}_{N}^{T}X_{i}^{*}\right)\right|\overset{P|F_{N}}{\to }0,$$

which can be proved as the same argument of Equation (A11) by Lagrange mean value theorem. Combine the bounds of Equations (A13) and (A14), one obtains

$$\max_{\gamma \in G(C_{0})}∥{\left[M_{n}\left({\hat{\beta }}_{N}\right)\right]}^{-1/2}V_{n}\left(\gamma \right){\left[M_{n}\left({\hat{\beta }}_{N}\right)\right]}^{-1/2}∥\overset{P|F_{N}}{\to }0.$$

Let δ∈(0,1) be a constant. Since $\underset{i\in S}{\sup}E(|e_{i}^{*}{|}^{1+\delta }|F_{N})<\infty$, one has

∑i∈SEei∗πi∗ψ¨β^NTXi∗Xi∗TMn(β^N)−1Xi∗1+δ|FN≤∑i∈SEei∗πi∗1+δ|FN·maxi∈Sψ¨β^NTXi∗1+δ·Xi∗TMn(β^N)−1Xi∗1+δ≤∑i∈S1πi∗1+δEei∗1+δ|FNmaxi∈Sψ¨β^NTXi∗1+δ·Xi∗T∑i∈SN1Nπi∗φ(^}βNTXi∗)Xi∗Xi∗T−1Xi∗1+δ=∑i∈S1Nπi∗1+δEei∗1+δ|FNmaxi∈Sψ¨β^NTXi∗1+δ·Xi∗T∑i∈S1Nπi∗φ(^}βNTXi∗)Xi∗Xi∗T−1Xi∗1+δ≤Cδ∑i∈SXi∗TX∗X∗T−1Xi∗1+δ≤Cδ∑i∈SXi∗TX∗X∗T−1Xi∗maxi∈SXi∗TX∗X∗T−1Xi∗δ=Cδmaxi∈SXi∗TX∗X∗T−1Xi∗δ∑i∈StrXi∗TX∗X∗T−1Xi∗=Cδmaxi∈SXi∗TX∗X∗T−1Xi∗δ∑i∈StrX∗X∗T−1Xi∗Xi∗T=Cδmaxi∈SXi∗TX∗X∗T−1Xi∗δtrX∗X∗T−1∑i∈SXi∗Xi∗T=Cδmaxi∈SXi∗TX∗X∗T−1Xi∗δtrX∗X∗T−1X∗X∗T=Cδmaxi∈SXi∗TX∗X∗T−1Xi∗δtrIp=pCδmaxi∈SXi∗TX∗X∗T−1Xi∗δ→P|FN0,

where $C_{\delta }>0$ is a constant. Under the definition of $W_{n}\left({\hat{\beta }}_{N}\right)$ and $E(e_{i}^{*}|F_{N})=0$, together with Theorem 1.14(ii) in [16], one obtains

$$∥{\left[M_{n}\left({\hat{\beta }}_{N}\right)\right]}^{-\frac{1}{2}}W_{n}\left({\hat{\beta }}_{N}\right){\left[M_{n}\left({\hat{\beta }}_{N}\right)\right]}^{-\frac{1}{2}}∥\overset{P|F_{N}}{\to }0.$$

Hence, Equation (A12) holds and the proof is completed. □

**Proof of** **Theorem 3.** According to the mean value theorem, one has

$$0=s_{n}\left({\hat{\beta }}_{n}\right)=s_{n}\left({\hat{\beta }}_{N}\right)+\nabla s_{n}\left(\bar{\bar{\beta }}\right)\left({\hat{\beta }}_{n}-{\hat{\beta }}_{N}\right),$$

where $\bar{\bar{\beta }}$ is between ${\hat{\beta }}_{n}$ and ${\hat{\beta }}_{N}$, then

(A15) $$\sqrt{n}\left({\hat{\beta }}_{n}-{\hat{\beta }}_{N}\right)=-\sqrt{n}\nabla s_{n}{\left(\bar{\bar{\beta }}\right)}^{-1}s_{n}\left({\hat{\beta }}_{N}\right).$$

Let $q_{i}^{*}\left({\hat{\beta }}_{N}\right)=\frac{1}{\pi_{i}^{*}}\left[Y_{i}^{*}-\mu \left(\psi \left({\hat{\beta }}_{N}^{T}X_{i}^{*}\right)\right)\right]\dot{\psi }\left({\hat{\beta }}_{N}^{T}X_{i}^{*}\right)X_{i}^{*}$, then

$$\begin{array}{cc} \sum_{i\in S}q_{i}^{*}\left({\hat{\beta }}_{N}\right) & =\sum_{i\in S}\frac{1}{\pi_{i}^{*}}\left[Y_{i}^{*}-\mu \left(\psi \left({\hat{\beta }}_{N}^{T}X_{i}^{*}\right)\right)\right]\dot{\psi }\left({\hat{\beta }}_{N}^{T}X_{i}^{*}\right)X_{i}^{*}=s_{n}\left({\hat{\beta }}_{N}\right). \end{array}$$

According to $E\left(Y_{i}^{*}|F_{N}\right)=\mu \left(\psi \left({\hat{\beta }}_{N}^{T}X_{i}^{*}\right)\right)$ in Equation (4), one obtains

$$E\left(q_{i}^{*}\left({\hat{\beta }}_{N}\right)|F_{N}\right)=\frac{1}{\pi_{i}^{*}}\left[E\left(Y_{i}^{*}|F_{N}\right)-\mu \left(\psi \left({\hat{\beta }}_{N}^{T}X_{i}^{*}\right)\right)\right]\dot{\psi }\left({\hat{\beta }}_{N}^{T}X_{i}^{*}\right)X_{i}^{*}=0.$$

Applying Lindeberg-L$\overset{´}{e}$vy CLT, one has

(A16) $$\frac{s_{n}\left({\hat{\beta }}_{N}\right)}{\sqrt{n}}\overset{d}{\to }N\left(0,\mathrm{Var}\left(q_{i}^{*}\left({\hat{\beta }}_{N}\right)|F_{N}\right)\right),$$

where

$$\begin{array}{cc} \mathrm{Var}(q_{i}^{*}\left({\hat{\beta }}_{N}\right)|F_{N})= & E(q_{i}^{*}\left({\hat{\beta }}_{N}\right)q_{i}^{*}{\left({\hat{\beta }}_{N}\right)}^{T}|F_{N})\\ = & \sum_{i\in U}\frac{a_{i}}{\pi_{i}}{\left[Y_{i}-\dot{b}\left(\psi \left({\hat{\beta }}_{N}^{T}X_{i}\right)\right)\right]}^{2}\dot{\psi }{\left({\hat{\beta }}_{N}^{T}X_{i}\right)}^{2}X_{i}X_{i}^{T}. \end{array}$$

Applying [26] in Theorem 2, one has

$$\frac{\nabla s_{n}\left(\bar{\bar{\beta }}\right)}{n}=\frac{1}{n}\sum_{i\in S}\frac{\partial q_{i}^{*}\left(\bar{\bar{\beta }}\right)}{\partial \bar{\bar{\beta }}}\overset{a.s.}{\to }E\left(\frac{\partial q_{i}^{*}\left(\bar{\bar{\beta }}\right)}{\partial \bar{\bar{\beta }}}|F_{N}\right),$$

where

$$\begin{array}{cc} E\left(\frac{\partial q_{i}^{*}\left(\bar{\bar{\beta }}\right)}{\partial \bar{\bar{\beta }}}|F_{N}\right)= & \sum_{i\in U}a_{i}\left[Y_{i}-\dot{b}\left(\psi \left({\bar{\bar{\beta }}}^{T}X_{i}\right)\right)\right]\ddot{\psi }\left({\bar{\bar{\beta }}}^{T}X_{i}\right)X_{i}X_{i}^{T}\\ & -\sum_{i\in U}a_{i}\ddot{b}\left(\psi \left({\bar{\bar{\beta }}}^{T}X_{i}\right)\right)\dot{\psi }{\left({\bar{\bar{\beta }}}^{T}X_{i}\right)}^{2}X_{i}X_{i}^{T}. \end{array}$$

Since $\bar{\bar{\beta }}$ is between ${\tilde{\beta }}_{n}$ and ${\hat{\beta }}_{N}$, and ${\tilde{\beta }}_{n}$ is consistent with ${\hat{\beta }}_{N}$ with respect to $F_{N}$ in probability, then

(A17) $$\frac{\nabla s_{n}\left(\bar{\bar{\beta }}\right)}{n}\overset{P|F_{N}}{\to }E\left(\frac{\partial q_{i}^{*}\left({\hat{\beta }}_{N}\right)}{\partial {\hat{\beta }}_{N}}|F_{N}\right),$$

where

$$\begin{array}{cc} E\left(\frac{\partial q_{i}^{*}\left({\hat{\beta }}_{N}\right)}{\partial {\hat{\beta }}_{N}}|F_{N}\right)= & \sum_{i\in U}a_{i}\left[Y_{i}-\dot{b}\left(\psi \left({\hat{\beta }}_{N}^{T}X_{i}\right)\right)\right]\ddot{\psi }\left({\hat{\beta }}_{N}^{T}X_{i}\right)X_{i}X_{i}^{T}\\ & -\sum_{i\in U}a_{i}\ddot{b}\left(\psi \left({\hat{\beta }}_{N}^{T}X_{i}\right)\right)\dot{\psi }{\left({\hat{\beta }}_{N}^{T}X_{i}\right)}^{2}X_{i}X_{i}^{T}. \end{array}$$

At last, combining Equations (A15)–(A17) by Slutsky’s theorem, one obtains

$$\sqrt{n}\left({\hat{\beta }}_{n}-{\hat{\beta }}_{N}\right)\overset{d}{\to }N\left(0,V_{s}\right),$$

where $V_{s}={\left[E\left(\frac{\partial q_{i}^{*}\left({\hat{\beta }}_{N}\right)}{\partial {\hat{\beta }}_{N}}|F_{N}\right)\right]}^{-1}\mathrm{Var}\left(q_{i}^{*}\left({\hat{\beta }}_{N}\right)|F_{N}\right){\left[E\left(\frac{\partial q_{i}^{*}\left({\hat{\beta }}_{N}\right)}{\partial {\hat{\beta }}_{N}}|F_{N}\right)\right]}^{-1}=\Sigma_{N}^{-1}V_{N}\Sigma_{N}^{-1}$. The proof is completed. □

**Proof of** **Theorem 4.** Here, one needs to prove the consistency of ${\hat{\beta }}_{n}$ with respect to $\beta_{0}$ due to the existence of ${\hat{\beta }}_{n}$; see [27]. Denote $p_{\beta }\left(X,y\right):=\exp\left\{y\psi \left(\beta^{T}X\right)-b\left(\psi \left(\beta^{T}X\right)\right)\right\}$, $m_{\beta }\left(X,y\right)=\log p_{\beta }\left(X,y\right):=$$y\psi \left(\beta^{T}X\right)-b\left(\psi \left(\beta^{T}X\right)\right)$ and $\tilde{\varphi }\left(\beta^{T}X\right)=\dot{b}\left[\psi \left(\beta^{T}X\right)\right]\dot{\psi }\left(\beta^{T}X\right)$. Then the negative K-L divergence in [28] is bounded,

$$\begin{array}{cc} -D_{KL}(P_{\beta_{0}}\left|\right|P_{\beta }):= & E_{\beta_{0}}\left(m_{\beta }-m_{\beta_{0}}\right)\\ = & E\{\left(E_{\beta_{0}}y|X\right)\left[\psi \left(\beta^{T}X\right)-\psi \left(\beta_{0}^{T}X\right)\right]-b\left(\psi \left(\beta^{T}X\right)\right)+b\left(\psi \left(\beta_{0}^{T}X\right)\right)\}\\ = & E\{\dot{b}\left[\psi \left(\beta_{0}^{T}X\right)\right]\left[\psi \left(\beta^{T}X\right)-\psi \left(\beta_{0}^{T}X\right)\right]-b\left(\psi \left(\beta^{T}X\right)\right)+b\left(\psi \left(\beta_{0}^{T}X\right)\right)\}\\ (\exists t_{1}\in \left[0,1\right])= & E\{\dot{b}\left[\psi \left(\beta_{0}^{T}X\right)\right]\left[\psi \left(\beta^{T}X\right)-\psi \left(\beta_{0}^{T}X\right)\right]\\ & -\dot{b}\left[\left(1-t_{1}\right)\psi \left(\beta^{T}X\right)+t_{1}\psi \left(\beta_{0}^{T}X\right)\right]\left[\psi \left(\beta^{T}X\right)-\psi \left(\beta_{0}^{T}X\right)\right]\}\\ (\exists t_{2}\in \left[0,1\right])= & E\{\ddot{b}\left[\left(1-t_{2}\right)\psi \left(\beta_{0}^{T}X\right)+\left(1-t_{1}\right)t_{2}\psi \left(\beta^{T}X\right)+t_{1}t_{2}\psi \left(\beta_{0}^{T}X\right)\right]\\ & \cdot \left[\psi \left(\beta_{0}^{T}X\right)-\left(1-t_{1}\right)\psi \left(\beta^{T}X\right)-t_{1}\psi \left(\beta_{0}^{T}X\right)\right]\left[\psi \left(\beta^{T}X\right)-\psi \left(\beta_{0}^{T}X\right)\right]\}\\ = & -\left(1-t_{1}\right)E\left\{\ddot{b}\left[\left(1-t_{3}\right)\psi \left(\beta_{0}^{T}X\right)+t_{3}\psi \left(\beta^{T}X\right)\right]{\left[\psi \left(\beta^{T}X\right)-\psi \left(\beta_{0}^{T}X\right)\right]}^{2}\right\}\\ (\exists t_{4}\in \left[0,1\right])= & -\left(1-t_{1}\right)E\{\ddot{b}\left[\left(1-t_{3}\right)\psi \left(\beta_{0}^{T}X\right)+t_{3}\psi \left(\beta^{T}X\right)\right]\\ & \cdot \dot{\psi }{\left[\left(1-t_{4}\right)\left(\beta_{0}^{T}X\right)+t_{4}\left(\beta^{T}X\right)\right]}^{2}{\left(\beta^{T}X-\beta_{0}^{T}X\right)}^{2}\}\\ \mathrm{By}\;\left(B.4\right)\;\mathrm{and}\;\left(B.5\right)]\leq & -\left(1-t_{1}\right)C_{1}E{\left(\beta^{T}X-\beta_{0}^{T}X\right)}^{2}\\ = & -\left(1-t_{1}\right)C_{1}{\left(\beta -\beta_{0}\right)}^{T}\left(EXX^{T}\right)\left(\beta -\beta_{0}\right)\\ \leq & -\left(1-t_{1}\right)C_{1}\lambda_{\min}\left(EXX^{T}\right)||\beta -\beta_{0}{\left|\right|}^{2}\\ \mathrm{By}\;\left(B.1\right)]\leq & -\left(1-t_{1}\right)C_{1}C_{2}||\beta -\beta_{0}{\left|\right|}^{2}, \end{array}$$

where $t_{3}=t_{2}-t_{1}t_{2}\in \left[0,1\right]$ and $C_{2}>0$. Then for any ε>0, one has the well-separation condition

$$\underset{||\beta -\beta_{0}{\left|\right|}^{2}\geq \varepsilon }{\sup}E_{\beta_{0}}m_{\beta }\left(X,y\right)<E_{\beta_{0}}m_{\beta_{0}}\left(X,y\right).$$

Let ${\tilde{M}}_{n}\left(\beta \right):=\frac{1}{n}\sum_{i=1}^{n}m_{\beta }\left(X_{i}^{*},Y_{i}^{*}\right)$, which is essentially a logarithmic likelihood function of subsampled GLMs, and ${\hat{\beta }}_{n}$ is the function’s maximum point. Thus, one has the nearly maximization ${\tilde{M}}_{n}\left({\hat{\beta }}_{n}\right)\geq {\tilde{M}}_{n}\left(\beta_{0}\right)\geq {\tilde{M}}_{n}\left(\beta_{0}\right)-o_{P}\left(1\right)$. Let $F:=\{m_{\beta }\left(X,y\right)=-y\psi \left(\beta^{T}X\right)+b\left(\psi \left(\beta^{T}X\right)\right),\beta \in \Theta \}$. Now one obtains

$$\begin{array}{cc} |m_{\beta_{1}}\left(X,y\right)-m_{\beta_{2}}\left(X,y\right)|= & |-y\psi \left(\beta_{1}^{T}X\right)+b\left(\psi \left(\beta_{1}^{T}X\right)\right)+y\psi \left(\beta_{2}^{T}X\right)-b\left(\psi \left(\beta_{2}^{T}X\right)\right)|\\ = & |y\psi \left(\beta_{1}^{T}X\right)-b\left(\psi \left(\beta_{1}^{T}X\right)\right)-y\psi \left(\beta_{2}^{T}X\right)+b\left(\psi \left(\beta_{2}^{T}X\right)\right)|\\ = & ||y\dot{\psi }\left(\xi_{\left(5\right)}^{T}X\right)\left(\beta_{1}^{T}X-\beta_{2}^{T}X\right)X\\ & -\dot{b}\left(\psi \left(\xi_{\left(6\right)}^{T}X\right)\right)\dot{\psi }\left(\xi_{\left(6\right)}^{T}X\right)\left(\beta_{1}^{T}X-\beta_{2}^{T}X\right)X\left|\right|\\ \leq & C_{4}|y-\dot{b}\left(\psi \left(\xi_{\left(6\right)}^{T}X\right)\right)\left|\cdot \right|\beta_{1}^{T}X-\beta_{2}^{T}X|\cdot ||X||\\ \leq & C_{4}|y-\dot{b}\left(\psi \left(\xi_{\left(6\right)}^{T}X\right)\right){\left|\cdot ||X|\right|}^{2}\cdot \left|\right|\beta_{1}-\beta_{2}\left|\right|,\forall \beta_{1},\beta_{2}\in \Theta , \end{array}$$

where $\xi_{\left(5\right)}$ and $\xi_{\left(6\right)}$ are both between $\beta_{1}$ and $\beta_{2}$ and $C_{4}>0$. Let $\bar{m}\left(X,y\right)=|y-\dot{b}\left(\psi \left(\xi_{\left(6\right)}^{T}X\right)\right){\left|\cdot ||X|\right|}^{2}$ and by (B.3), one has

$$\left|\right|\bar{m}{\left(X,y\right)||}_{P,1}:=E_{\beta_{0}}|\bar{m}\left(X,Y\right)|\leq E_{\beta_{0}}\underset{\forall \beta \in \Theta }{\sup}[|y-\dot{b}\left(\psi \left(\beta^{T}X\right)\right){\left|\cdot ||X|\right|}^{2}]<\infty ,$$

where $\left|\right|\cdot {\left|\right|}_{\tilde{P},1}=\tilde{P}\left|\cdot \right|$ is the $L_{1}\left(\tilde{P}\right)$-norm in P269-P270 of [11] and $\tilde{P}:=E_{\beta_{0}}$. And then from the Example 19.7 in [11], one obtains

$$N_{\left[\;\right]}\left(\varepsilon ,F,L_{1}\left(E_{\beta_{0}}\right)\right)\leq K{\left(\frac{\mathrm{diam}\Theta }{\varepsilon /\left|\right|\bar{m}{\left|\right|}_{E_{\beta_{0}},1}}\right)}^{p}<\infty ,\;\mathrm{every}\;0<\varepsilon <\mathrm{diam}\Theta <\infty$$

where $N_{\left[\;\right]}\left(\varepsilon ,F,L_{1}\left(E_{\beta_{0}}\right)\right)$ is called bracketingnumber which is the minimum number of ε-brackets needed to cover F; see P270 in [11]. And *K* is a constant, and $\mathrm{diam}\Theta =\sup_{\forall \beta_{1},\beta_{2}\in \Theta }\left|\right|\beta_{1}-\beta_{2}\left|\right|$. Therefore, the class F is P-Glivenko-Cantelli by Theorem 19.4 in [11]. And from the definition of P-Glivenko-Cantelli in P269 of [11], we have

$$\underset{\forall \beta \in \Theta }{\sup}\left|{\tilde{M}}_{n}\left(\beta \right)-E_{\beta_{0}}m_{\beta }\left(X,y\right)\right|\overset{a.s.}{\to }0.$$

Finally, according to Theorem 5.7 in [11], we get $∥{\hat{\beta }}_{n}-\beta_{0}∥=o_{P}\left(1\right)$. The proof is then completed. □

Recall (A7) and (A8) respectively, then $\nabla s_{n}\left(\gamma \right)=R_{n}\left(\gamma \right)-M_{n}\left(\gamma \right)$. Let $\Phi =E(\nabla s_{n}\left(\beta \right))$, then we have the following lemma.

**Lemma** **A3.**
*For $\beta \in R^{p}$, assume that*

(E.1)  $R_{n}\left(\beta \right)$ *is finite and nonsingular*.
(E.2) *For 1≤k,j≤p,*
  $$E{\left[\sum_{i\in S}\frac{a_{i}}{\pi_{i}}\left[Y_{i}-\mu \left(\psi \left(\beta^{T}X_{i}\right)\right)\right]\ddot{\psi }\left(\beta^{T}X_{i}\right)x_{ik}x_{ij}\right]}^{2}=o\left(1\right).$$
(E.3) *For 1≤k,j≤p,*
  $$\mathrm{Var}\left[\sum_{i\in U}\frac{a_{i}}{\pi_{i}}{\left[\dot{\psi }\left(\beta^{T}X_{i}\right)\right]}^{2}\dot{\mu }\left(\psi \left(\beta^{T}X_{i}\right)\right)x_{ik}x_{ij}\right]=o\left(1\right).$$
  *Then,*
  $$\nabla s_{n}\left(\beta \right)\to \Phi .$$

**Proof.** One derives each entry in the matrix by

$$\begin{array}{cc} {\left(\nabla s_{n}\left(\beta \right)\right)}_{kj}= & {\left(R_{n}\left(\beta \right)\right)}_{kj}-{\left(M_{n}\left(\beta \right)\right)}_{kj}\\ = & \sum_{i\in S}\frac{1}{\pi_{i}^{*}}\left[Y_{i}^{*}-\mu \left(\psi \left(\beta^{T}X_{i}^{*}\right)\right)\right]\ddot{\psi }\left(\beta^{T}X_{i}^{*}\right)x_{ik}^{*}x_{ij}^{*}\\ & -\sum_{i\in S}\frac{1}{\pi_{i}^{*}}{\left[\dot{\psi }\left(\beta^{T}X_{i}^{*}\right)\right]}^{2}\dot{\mu }\left(\psi \left(\beta^{T}X_{i}^{*}\right)\right)x_{ik}^{*}x_{ij}^{*}. \end{array}$$

By the definition of Φ, one has

$$\Phi_{kj}=E{\left(\nabla s_{n}\left(\beta \right)\right)}_{kj}=E\left[-\sum_{i\in S}\frac{1}{\pi_{i}^{*}}\dot{\mu }\left(\psi \left(\beta^{T}X_{i}^{*}\right)\right){\left[\dot{\psi }\left(\beta^{T}X_{i}^{*}\right)\right]}^{2}x_{ik}^{*}x_{ij}^{*}\right].$$

Next, one obtains

$$\begin{array}{cc} & E{\left[{\left(\nabla s_{n}\left(\beta \right)\right)}_{kj}-\Phi_{kj}\right]}^{2}\\ = & E\left\{{\left[{\left(\nabla s_{n}\left(\beta \right)\right)}_{kj}-\Phi_{kj}\right]}^{2}|{\left(X_{i},Y_{i}\right)}_{i=1}^{N}\right\}\\ = & E\left\{{\left[{\left(\nabla s_{n}\left(\beta \right)\right)}_{kj}-E{\left(\nabla s_{n}\left(\beta \right)\right)}_{kj}\right]}^{2}|{\left(X_{i},Y_{i}\right)}_{i=1}^{N}\right\}\\ = & E\left\{{\left[{\left(R_{n}\left(\beta \right)\right)}_{kj}-{\left(M_{n}\left(\beta \right)\right)}_{kj}+E{\left(M_{n}\left(\beta \right)\right)}_{kj}\right]}^{2}|{\left(X_{i},Y_{i}\right)}_{i=1}^{N}\right\}\\ = & E\left\{{\left(R_{n}\left(\beta \right)\right)}_{kj}^{2}+{\left[E{\left(M_{n}\left(\beta \right)\right)}_{kj}-{\left(M_{n}\left(\beta \right)\right)}_{kj}\right]}^{2}|{\left(X_{i},Y_{i}\right)}_{i=1}^{N}\right\}\\ & +E\left\{2{\left(R_{n}\left(\beta \right)\right)}_{kj}\left[E{\left(M_{n}\left(\beta \right)\right)}_{kj}-{\left(M_{n}\left(\beta \right)\right)}_{kj}\right]|{\left(X_{i},Y_{i}\right)}_{i=1}^{N}\right\}\\ = & E\left\{{\left(R_{n}\left(\beta \right)\right)}_{kj}^{2}|{\left(X_{i},Y_{i}\right)}_{i=1}^{N}\right\}+\mathrm{Var}\left\{{\left(M_{n}\left(\beta \right)\right)}_{kj}|{\left(X_{i},Y_{i}\right)}_{i=1}^{N}\right\}\\ = & o(1), \end{array}$$

where the first equality is based on the fact that after conditioning on the *N* data points, the *n* repeating sampling steps should be independent and identically distributed in each step. The last equality holds by the conditions (E.2) and (E.3). □

**Lemma** **A4.**
*Under the conditions (C.1)–(C.5) in Theorem 5, if $s_{n}\left({\hat{\beta }}_{n}\right)=0$ for all large n and $\left|\right|{\hat{\beta }}_{n}-\beta_{0}\left|\right|=O_{P}\left(1/N\right)$, then*

$$s_{n}\left(\beta_{0}\right)=-\Phi \left({\hat{\beta }}_{n}-\beta_{0}\right)+o_{P}\left(1\right).$$

**Proof.** By Taylor’s expansion:

$$0=s_{n}\left({\hat{\beta }}_{n}\right)=s_{n}\left(\beta_{0}\right)+\nabla s_{n}\left(\beta_{0}\right)\left({\hat{\beta }}_{n}-\beta_{0}\right)+\frac{1}{2}{\left({\hat{\beta }}_{n}-\beta_{0}\right)}^{T}\Sigma \left({\tilde{\beta }}_{n}\right)\left({\hat{\beta }}_{n}-\beta_{0}\right),$$

where $\Sigma \left({\tilde{\beta }}_{n}\right)=\nabla^{2}s_{n}\left({\tilde{\beta }}_{n}\right)$ and ${\tilde{\beta }}_{n}$ is between $\beta_{0}$ and ${\hat{\beta }}_{n}$. From assumption (C.3), (C.4) and (C.5) in Theorem 5, we have

$$\begin{array}{cc} ∥\Sigma ({\tilde{\beta }}_{n})∥ & =∥\sum_{i\in S}\frac{1}{\pi_{i}^{*}}{\ddot{\phi }}_{\beta }\left(X_{i}^{*},Y_{i}^{*}\right)∥\\ & \leq \sum_{i\in S}\frac{1}{\pi_{i}^{*}}\cdot ∥{\ddot{\phi }}_{\beta }\left(X_{i}^{*},Y_{i}^{*}\right)∥=O\left(nN\right). \end{array}$$

Then $\frac{1}{2}{\left({\hat{\beta }}_{n}-\beta_{0}\right)}^{T}\Sigma \left({\tilde{\beta }}_{n}\right)\left({\hat{\beta }}_{n}-\beta_{0}\right)=o_{P}\left(1\right)$. Therefore, by Lemma A3, one has

$$0=s_{n}\left(\beta_{0}\right)+\left(\Phi +o\left(1\right)\right)\left({\hat{\beta }}_{n}-\beta_{0}\right)+o_{P}\left(1\right),$$

which implies

$$s_{n}\left(\beta_{0}\right)=-\Phi \left({\hat{\beta }}_{n}-\beta_{0}\right)+o_{P}\left(1\right).$$

Hence, the proof is completed. □

**Lemma** **A5.**
*${\left\{{\bar{M}}_{i}\right\}}_{i=1}^{n}$ is a martingale difference sequence adapt to the filtration ${\left\{F_{N,i}\right\}}_{i=1}^{n}$.*

**Proof.** The ${\bar{M}}_{i}$’s are $F_{N,i}$-measurable by the definition of ${\bar{M}}_{i}$ and the definition of the filtration ${\left\{F_{N,i}\right\}}_{i=1}^{n}$. Then we obtain

$$\begin{array}{cc} E[{\bar{M}}_{i}|F_{N,i-1}] & =E\left[\frac{1}{\pi_{i}^{*}}\phi_{\beta }\left(X_{i}^{*},Y_{i}^{*}\right)-\sum_{j=1}^{N}\phi_{\beta }\left(X_{j},Y_{j}\right)|F_{N,i-1}\right]\\ & =E\left[\frac{1}{\pi_{i}^{*}}\phi_{\beta }\left(X_{i}^{*},Y_{i}^{*}\right)|F_{N,i-1}\right]-E\left[\sum_{j=1}^{N}\phi_{\beta }\left(X_{j},Y_{j}\right)|F_{N,i-1}\right]\\ & =\frac{\sum_{i=1}^{N}\pi_{i}\frac{1}{\pi_{i}}\phi_{\beta }\left(X_{i},Y_{i}\right)}{\sum_{i=1}^{N}\pi_{i}}-\frac{\sum_{i=1}^{N}\pi_{i}\sum_{j=1}^{N}\phi_{\beta }\left(X_{j},Y_{j}\right)}{\sum_{i=1}^{N}\pi_{i}}\\ & =\sum_{i=1}^{N}\phi_{\beta }\left(X_{i},Y_{i}\right)-\sum_{j=1}^{N}\phi_{\beta }\left(X_{j},Y_{j}\right)\\ & =0. \end{array}$$

By the definition of martingale difference sequence in P230 of [29], the proof is completed. □

Under the definition of $T,\bar{M},Q$, it is obvious that $\mathrm{Var}\left(T\right)=\mathrm{Var}\left(\bar{M}\right)+\mathrm{Var}\left(Q\right)$.

**Lemma** **A6.**
*$\underset{N}{\sup}\lambda_{\max}\left(B_{N}\right)\leq 1$.*

**Proof.** By symmetry of $B_{N}$, we only to show for any *N*, $I-B_{N}$ is positive definite.

$$\begin{array}{cc} I-B_{N} & =\mathrm{Var}{\left(T\right)}^{-\frac{1}{2}}\left(\mathrm{Var}\left(T\right)-\mathrm{Var}\left(\bar{M}\right)\right)\mathrm{Var}{\left(T\right)}^{-\frac{1}{2}}\\ & =\mathrm{Var}{\left(T\right)}^{-\frac{1}{2}}\mathrm{Var}\left(Q\right)\mathrm{Var}{\left(T\right)}^{-\frac{1}{2}}. \end{array}$$

Therefore, $I-B_{N}$ is equivalent to the positive definite matrix Var(Q). The proof is completed. □

**Lemma** **A7** (Multivariate version of martingale CLT, Lemma 4 in [19])**.** *For k=1,2,3,…, let $\left\{\xi_{ki};i=1,2,\ldots ,N_{k}\right\}$ be a martingale difference sequence in $R^{p}$ relative to the filtration $\left\{F_{ki};i=0,1,\ldots ,N_{k}\right\}$ and let $Y_{k}\in R^{p}$ be an $F_{k0}$-measurable random vector. Set $S_{k}=\sum_{i=1}^{N_{k}}\xi_{ki}$. Assume that*

(F.1) *$\lim_{k\to \infty }\sum_{i=1}^{N_{k}}E[∥\xi_{ki}{∥}^{4}]=0$;*
(F.2) *$\lim_{k\to \infty }E\left[{∥\sum_{i=1}^{N_{k}}\left[\xi_{ki}\xi_{ki}^{T}|F_{k,i-1}\right]-B_{k}∥}^{2}\right]=0$ for some sequence of positive definite matrices ${\left\{B_{k}\right\}}_{k=1}^{\infty }$ with $\underset{k}{\sup}\lambda_{max}\left(B_{k}\right)<\infty$ i.e., the largest eigenvalue is uniformly bounded;*
(F.3) *For some probability distribution $L_{0}$, ∗ denotes convolution and L(·) denotes the law of random variates:*
  $$L\left(Y_{k}\right)*N\left(0,B_{k}\right)\overset{d}{\to }L_{0}.$$
  *Then*
  $$L\left(Y_{k}+S_{k}\right)\overset{d}{\to }L_{0}.$$

**Lemma** **A8** (Asymptotic normality of $s_{n}\left(\beta_{0}\right)$)**.** *Assume that*

(G.1.) *$\lim_{N\to \infty }\sum_{i=1}^{n}E[||\xi_{Ni}{\left|\right|}^{4}]=0$;*
(G.2.) *$\lim_{N\to \infty }E\left[{∥\sum_{i=1}^{n}E\left[\xi_{Ni}\xi_{Ni}^{T}|F_{N,i-1}\right]-B_{N}∥}^{2}\right]=0$.*
  *Then*
  $$\mathrm{Var}{\left(T\right)}^{-\frac{1}{2}}\cdot T\overset{d}{\to }N\left(0,I_{p}\right).$$

**Proof.** The conditions in lemma A7 can be substituted with

$$\begin{array}{cc} & \xi_{ki}=\xi_{Ni},\;Y_{k}=\mathrm{Var}{\left(T\right)}^{-\frac{1}{2}}\cdot Q,\\ & B_{k}=B_{N},\;L_{0}∼N\left(0,I_{p}\right). \end{array}$$

By Lemma A5, conditions (F.1) and (F.2) of Lemma A7 are satisfied. Next we only need to show the third condition in Lemma A7 holds. According to central limit theorem we have

$$\mathrm{Var}{\left(Q\right)}^{-\frac{1}{2}}\cdot Q\overset{d}{\to }N\left(0,I_{p}\right).$$

For any $t\in R^{p}$, let $\tilde{t}=\mathrm{Var}{\left(T\right)}^{-\frac{1}{2}}t$, $\tilde{X}=iQ$, due to the properties of the complex multivariate normal distributions are equivalent to the properties of real multivariate normal distributions in P222 of [30], and EQ=0, one has

$$\begin{array}{cc} \mathrm{Var}(\tilde{X}) & =\mathrm{Var}\left(iQ\right)=E\left[\left(iQ\right)\left(iQ\right)\right]-{\left[E\left(iQ\right)\right]}^{2}\\ & =-EQ^{2}=-EQ^{2}+{\left(EQ\right)}^{2}=-\mathrm{Var}\left(Q\right). \end{array}$$

Thus, according to Equations (45.4)–(45.6) in P108 of [30], one has

$$Ee^{{\tilde{t}}^{T}\tilde{X}}=e^{{\tilde{t}}^{T}E\left(\tilde{X}\right)+\frac{1}{2}{\tilde{t}}^{T}\mathrm{Var}\left(\tilde{X}\right)\tilde{t}}=e^{-\frac{1}{2}t^{T}\mathrm{Var}{\left(T\right)}^{-\frac{1}{2}}\mathrm{Var}\left(Q\right)\mathrm{Var}{\left(T\right)}^{-\frac{1}{2}}t}.$$

Further, we obtain

$$E\left[e^{it^{T}\mathrm{Var}{\left(T\right)}^{-\frac{1}{2}}Q}\right]\cdot e^{-\frac{1}{2}t^{T}\mathrm{Var}{\left(T\right)}^{-\frac{1}{2}}\mathrm{Var}\left(\bar{M}\right)\mathrm{Var}{\left(T\right)}^{-\frac{1}{2}}t}=e^{-\frac{1}{2}t^{T}t}.$$

Therefore, condition (F.3) in Lemma A7 is verified. Then one obtains

$$\mathrm{Var}{\left(T\right)}^{-\frac{1}{2}}T=\mathrm{Var}{\left(T\right)}^{-\frac{1}{2}}\cdot Q+\mathrm{Var}{\left(T\right)}^{-\frac{1}{2}}\cdot \bar{M}\overset{d}{\to }N\left(0,I_{p}\right).$$

The proof is completed. □

**Proof of** **Theorem 5.** According to Lemma A4,

(A18) $$\Phi \left({\hat{\beta }}_{n}-\beta_{0}\right)+o_{P}\left(1\right)=-s_{n}\left(\beta_{0}\right)=-T.$$

Multiplying with $\mathrm{Var}{\left(T\right)}^{-\frac{1}{2}}$ in (A18), one obtains

$$\mathrm{Var}{\left(T\right)}^{-\frac{1}{2}}\Phi \left({\hat{\beta }}_{n}-\beta_{0}\right)+o_{P}(||\mathrm{Var}{\left(T\right)}^{-\frac{1}{2}}\left||\right)=-\mathrm{Var}{\left(T\right)}^{-\frac{1}{2}}T.$$

Applying Lemma A8, one obtains

$$\mathrm{Var}{\left(T\right)}^{-\frac{1}{2}}\Phi \left({\hat{\beta }}_{n}-\beta_{0}\right)\overset{d}{\to }N\left(0,I_{p}\right).$$

The proof is completed. □
